# Glycemic control, HbA1c variability, and major cardiovascular adverse outcomes in type 2 diabetes patients with elevated cardiovascular risk: insights from the ACCORD study

**DOI:** 10.1186/s12933-023-02026-9

**Published:** 2023-10-27

**Authors:** Junyu Pei, Xiaopu Wang, Zeyu Pei, Xinqun Hu

**Affiliations:** 1 Department of Cardiovascular Medicine, The Second Xiangya Hospital, Central South University, Changsha, Hunan China; 2 The Libin Cardiovascular Institute of Alberta, Cumming school of Medicine, The University of Calgary, Calgary, AB Canada; 3 China Power International Development Limited, Hong Kong, China

**Keywords:** HbA1c variability, Intensive blood glucose treatment, Mean HbA1c, Machine learning algorithms

## Abstract

**Background:**

Although recent guidelines advocate for HbA1c target individualization, a comprehensive criterion for patient categorization remains absent. This study aimed to categorize HbA1c variability levels and explore the relationship between glycemic control, cardiovascular outcomes, and mortality across different degrees of variability.

**Methods:**

Action to Control Cardiovascular Risk in Diabetes study data were used. HbA1c variability was measured using the HbA1c variability score (HVS) and standard deviation (SD). K-means and K-medians clustering were used to combine the HVS and SD.

**Results:**

K-means clustering was the most stable algorithm with the lowest clustering similarities. In the low variability group, intensive glucose-lowering treatment significantly reduced the risk of adverse cardiovascular outcomes (HR: 0·78 [95% CI: 0·63, 0·97]) without increasing mortality risk (HR: 1·07 [0.81, 1·42]); the risk of adverse cardiovascular events (HR: 1·33 [1·14, 1·56]) and all-cause mortality (HR: 1·23 [1·01,1·51]) increased with increasing mean HbA1c. In the high variability group, treatment increased the risk of cardiovascular events (HR: 2.00 [1·54, 2·60]) and mortality (HR: 2·20 [1·66, 2·92]); a higher mean HbA1c (7·86%, [7·66%, 8·06%]) had the lowest mortality risk, when the mean HbA1c was < 7·86%, a higher mean HbA1c was associated with a lower mortality risk (HR: 0·63 [0·42, 0·95]). In the medium variability group, a mean HbA1c around 7·5% was associated with the lowest risk.

**Conclusions:**

HbA1c variability can guide glycemic control targets for patients with type 2 diabetes. For patients with low variability, the lower the HbA1c, the lower the risk. For those with medium variability, controlling HbA1c at 7·5% provides the maximum benefit. For patients with high variability, a mean HbA1c of around 7·8% presents the lowest risk of all-cause mortality, a lower HbA1c did not provide cardiovascular benefits but instead increased the mortality risk. Further studies, especially those with patients that reflect the general population with type 2 diabetes undergoing the latest therapeutic approaches, are essential to validate the conclusions of this study.

**Supplementary Information:**

The online version contains supplementary material available at 10.1186/s12933-023-02026-9.

## Introduction

Type 2 diabetes mellitus (T2DM) patients with hemoglobin A1c (HbA1c) values in the normal range (< 6·0%) had the lowest risk of cardiovascular events [1]. The goal of treatment of patients with T2DM is to reduce blood glucose levels, which can be attained through conventional and intensive glucose-lowering management, but this has also been strongly associated with adverse outcomes [1–3]. The conclusions of recent randomized controlled trials that studied the effectiveness of intensive blood glucose management have not been consistent or conclusive [4–9].

In addition to the level of glycemia, studies have recently identified glycemic variability as a potential risk factor for adverse outcomes in people with T2DM [10]. Most studies evaluated HbA1c variability using the standard deviation (SD) or the coefficient of variation (CV) of HbA1c [11–13]. However, both the SD and CV of HbA1c were difficult to interpret in clinical practice. In 2018, Forbes et al. proposed a new method to evaluate HbA1c variability, namely the HbA1c variability score (HVS). The HVS was calculated as the percentage of HbA1c level changes > 0·5% (5·5 mmol/mol) among all HbA1c measurements for an individual. This measure of HbA1c was much more clinically translatable.

Recent studies have also demonstrated that patients with T2DM with “low and stable” patterns of HbA1c over time have lower risks [14, 15]. However, the relationship between HbA1c variability and blood glucose-lowering targets has not been well-studied. Thus, we used machine learning algorithms to cluster the HbA1c variability of participants into low, medium, and high levels based on the HVS and SD of HbA1c; this study aimed to investigate the optimal target of glucose-lowering treatment for patients with different HbA1c variability.

## Methods

### Study participants and data collection

We used the data from the Action to Control Cardiovascular Risk in Diabetes (ACCORD) study to perform a *post-hoc* analysis. The ACCORD trial was specifically designed to determine whether a therapeutic strategy targeting the normalization of glycated hemoglobin levels (i.e., below 6·0%) would reduce the rate of cardiovascular events, compared with a strategy targeting glycated hemoglobin levels from 7·0 to 7·9% in a population of middle-aged and older people with T2DM and either established cardiovascular disease or additional cardiovascular risk factors [4]. We obtained the ACCORD data from the Biologic Specimen and Data Repository Information Coordinating Center BioLINCC, National Heart, Lung, and Blood Institute, U.S. Department of Health & Human Services. The data can be accessed upon reasonable request at https://biolincc.nhlbi.nih.gov/studies/accord/. The design of the ACCORD study and its main results have been published previously [4, 16–18]. Patients included in the study had a mean age of 62 years, a mean 10-year history of T2DM, and a mean glycated HbA1c level of 8·3%. Study results showed that intensive glycemic control did not reduce death or nonfatal cardiovascular events, but did increase all-cause mortality. Given these results, intensive control of blood glucose was halted after a mean follow-up of 3·7 years because it increased the risk of all-cause mortality.

### Exposure variables and study outcome

This study measured HbA1c variability using the HVS and SD. The HVS was calculated as the percentage of the number of changes in HbA1c > 0·5% (5·5 mmol/mol) among all HbA1c measurements within an individual. SD was defined as the arithmetic square root of the arithmetic means square of the square of all glycosylated hemoglobin measurements within an individual. The primary outcome was major cardiovascular adverse events (MACEs), which was defined as a composite outcome of nonfatal myocardial infarction, nonfatal stroke, or death from cardiovascular causes. The secondary outcome was all-cause mortality. And the hypoglycemic events defined as patients occurred hypoglycemia requiring any assistance.

### Statistical analysis

Participants’ baseline characteristics and crude outcomes were presented as means and SDs or medians and interquartile ranges for continuous variables and as frequencies and percentages for categorical variables depending on whether the datasets were normally distributed. We compared the participants’ baseline characteristics using the chi-square or Mann–Whitney U test according to each variable’s distribution type.

We conducted various analyses adjusted for several factors, including age, sex, ethnicity, body mass index (BMI), blood pressure, hyperlipidemia, estimated glomerular filtration rate, comorbidity (heart failure, depression, albuminuria), and smoking. A generalized additive model (GAM) was used to reveal the non-linear association of HbA1c variability and mean HbA1c with the hazard ratios (HRs) of the outcomes. The proportional hazard assumption appeared to be violated for the secondary outcome; thus, we used the Weibull accelerated failure time model, which was an alternative strategy for the analysis of time-to-event data and was suitable for estimating the HRs of the primary outcome and all-cause mortality even when the hazards were not proportional [19]. The Akaike Information Criterion (AIC), Bayesian Information Criterion (BIC), and residuals indicated that the Weibull accelerated failure time model better fit the data. We then used the log likelihood ratio test to detect the interaction effect between HbA1c variability and different glucose-lowering treatment strategies. Subsequently, we used the two-piecewise linear-regression model and bootstrap method to calculate the threshold effect of the relationship between the mean HbA1c and the outcomes.

To compare with the quantile clustering, two unsupervised machine learning algorithms, via K-means and K-medians clustering, were used to cluster HbA1c variability by examining both the HVS and the SD. K-means clustering is a method of vector quantization, originally from signal processing, that aims to partition “n” observations into “k” clusters in which each observation belongs to the cluster with the nearest mean (cluster centers or centroid), serving as a prototype of the cluster. K-medians clustering is a variation of K-means clustering where instead of calculating the mean for each cluster to determine its centroid, one is used to calculate the median. This minimizes error over all clusters concerning the one-norm distance metric, as opposed to the squared two-norm distance metric (as in K-means). Three internal validation metrics (Calinski-Harabaz, Davies-Bouldin, and Silhouette Indexes) were used to describe the data dispersion within and across the clusters and to compute the ratio of similarity of the original data from the clusters, respectively. Low values of the Davies-Bouldin Index and high values of the Calinski-Harabaz and Silhouette Indexes reflected better clustering with less similarity, so the clusters of data show different characteristics. We calculated the internal validation metrics separately when dividing the total population into k (k = 2–9) groups; after combining the two metrics (Davies-Bouldin and Silhouette Indexes), we concluded that clustering performed best when dividing the population into three groups (Table S1). Patients were then clustered into three equal-sized groups of low, medium, or high HbA1c variability levels with the quantile clustering on the HVS or SD. Participants with a high level of HbA1c variability were identified as the top one-third of the population.

### Sensitivity analysis

We performed a sensitivity analysis to test the relationship between HbA1c variability and the primary and secondary outcomes by excluding participants who had a follow-up time of less than 1 year because these patients might have had unknown diseases. Moreover, according to the ACCORD study protocol, participants would have at least four HbA1c results in the first year of follow-up [18]. To further ascertain the robustness of our findings, we conducted a sensitivity analysis using variation independent of the mean (VIM) as an alternative to the SD [20]. The calculation formula for VIM is as follows:

$$\eqalign{VIM & = Population\,mea{n^x}*\frac{{SD}}{{mea{n^x}}}\cr & (x\,derived\;from\,curve\,fitting)}$$

We performed all analyses using Stata 16·0, Python version 3·6·0, and R Version 4·0·2. *P*-values < 0·05 (two-sided) were considered statistically significant.

## Results

### Baseline characteristics

The ACCORD study enrolled 10,251 participants, of which 5,128 were randomized to achieve a target glycated hemoglobin level < 6·0% (intensive treatment group). We excluded 199 participants whose HVS could not be calculated. Hence, our *post-hoc* analysis included 10,052 participants. The mean age of the participants was 62·7 years (SD 6·62), and 6,196 (61·6%) of the participants were men. After a median follow-up of 4·82 years, MACEs occurred in 1,015 participants, and all-cause mortality occurred in 668 participants. The baseline characteristics and crude outcomes of the included participants are presented in Table 1. Under different variability groupings, the baseline characteristics between the intensive management group and the standard management group were essentially consistent (Table S2).

**Table 1:** Baseline characteristics and crude endpoints of the study participants

K-means Variability Group	Low	Medium	High	*P*-value
N	4489	3929	1634	
Age	63.6 ± 6.6	62.3 ± 6.5	61.5 ± 6.6	< 0.01
Female	1697 (37.8%)	1499 (38.2%)	660 (40.4%)	0.17
Race or ethnic group (%)				< 0.01
White	3002 (66.9%)	2417 (61.5%)	862 (52.8%)	
Black	668 (14.9%)	781 (19.9%)	455 (27.8%)	
Hispanic	250 (5.6%)	296 (7.5%)	173 (10.6%)	
Other	569 (12.7%)	435 (11.1%)	144 (8.8%)	
CVD History (%)	1446 (32.2%)	1432 (36.4%)	638 (39%)	< 0.01
Education (%)				< 0.01
Less than high school	576 (12.8%)	602 (15.3%)	286 (17.5%)	
High-school graduate	1180 (26.3%)	1038 (26.4%)	444 (27.2%)	
Some college	1466 (32.7%)	1302 (33.1%)	530 (32.5%)	
College degree or higher	1264 (28.2%)	986 (25.1%)	766 (23.3%)	
Intensive Treatment (%)	2829 (63.0%)	1624 (41.3%)	574 (35.1%)	< 0.01
HbA_1c_ measures				
Baseline HbA_1C_ (%)	8.0 ± 0.9	8.5 ± 1.1	8.8 ± 1.2	< 0.01
HVS	19.3 ± 8.9	45.4 ± 8.3	75.9 ± 13.0	< 0.01
SD of HbA_1C_	0.5 ± 0.2	0.8 ± 0.3	1.1 ± 0.4	< 0.01
Blood pressure (mm Hg)				
Systolic	135.8 ± 16.3	136.5 ± 17.4	137.2 ± 18.1	0.04
Diastolic	74.4 ± 10.2	74.9 ± 10.8	76.1 ± 11.3	< 0.01
Heart rate (BPM)	72.0 ± 11.5	72.8 ± 11.7	74.1 ± 12.2	< 0.01
Body-mass index	31.8 ± 5.2	32.5 ± 5.5	32.8 ± 5.6	< 0.01
Cholesterol (mg/dl)				
Total	181.0 ± 39.7	184.8 ± 43.1	186.2 ± 44.0	< 0.01
Low-density lipoprotein	103.5 ± 32.6	105.6 ± 34.2	106.8 ± 36.2	< 0.01
High-density lipoprotein	42.3 ± 11.6	41.5 ± 11.4	41.7 ± 12.1	< 0.01
Triglyceride (mg/dl)	182.1 ± 125.4	195.9 ± 157.1	199.8 ± 183.3	< 0.01
Fasting serum glucose (mg/dl)	167.4 ± 47.9	179.2 ± 58.3	187.7 ± 67.9	< 0.01
Estimated GFR, mL *min^− 1^*1.73 m^− 2^	90.5 ± 24.4	91.3 ± 30.3	92.2 ± 26.1	0.01
History physical exam (%)				
Protein in urine	788 (17.6%)	833 (21.2%)	373 (21.8%)	< 0.01
Heart failure	173 (3.9%)	202 (5.1%)	99 (6.1%)	< 0.01
Neuropathy	1093 (24.4%)	1099 (28.0%)	485 (29.7%)	< 0.01
Depression	893 (19.9%)	995 (25.3%)	482 (29.5%)	< 0.01
Eye disease	1367 (30.5%)	1266 (32.2%)	506 (31.0%)	0.10
Smoked cigarettes in last 30 days	539 (12.0%)	564 (14.4%)	289 (17.7%)	< 0.01
Crude outcomes (%)				
Primary outcome	340 (7.6%)	442 (11.2%)	233 (14.3%)	< 0.01
All-cause mortality	216 (4.8%)	254 (6.5%)	198 (12.4%)	< 0.01
CVD-mortality	93 (2.1%)	118 (3.0%)	95 (5.8%)	< 0.01
Non-fatal MI	213 (4.7%)	293 (7.5%)	118 (7.2%)	< 0.01
Non-fatal Stroke	56 (1.2%)	68 (1.7%)	49 (3.0%)	< 0.01
Total Stroke	63(1.4%)	77(2.0%)	52(3.2%)	< 0.01

HVS, HbA1c variability score; CVD, cardiovascular diseases; GFR, glomerular filtration rate; MI, myocardial infarction

### Clustering results

Patients who received intensive treatment had a relatively lower HVS and higher SD (Table S3). We classified all participants into three groups (low, medium, and high HbA1c variability). Quantile partitioning is the traditional method of clustering participants into three equal groups of the population (Table S4). Two machine learning algorithms created a smaller group of participants with a high level of HbA1c variability: 16·3% by K-means and 24·4% by K-medians. Quantile partitioning clusters were purely based on HbA1c record variation, while the machine learning model considered both the HVS and the SD (Figure S1). In the low, medium, and high variability groups, the mean values of HVS are 19.29, 45.44, and 75.95, respectively, while the mean values of SD are also 0.53, 0.77, and 1.06, respectively (K-means). Cut-off values for the HVS and SD among the clustering methods were comparable (Table S3, S4). In the high variability group, the effect of intensive blood glucose control was the lowest and relatively increased HbA1c variability. Conversely, in the low variability group, intensive blood glucose control had the best glucose-lowering effect and significantly reduced blood HbA1c variability. All three evaluation indicators for the clustering methods showed that the K-means method had the best performance (Davies-Bouldin Index: 0·570, Silhouette Index: 0·523, Calinski-Harabaz Index: 23,041·551) (Table S5).

### HbA1c variability and outcomes

Increased HbA1c variability significantly increased the risk of adverse outcomes. The risk of MACEs and all-cause mortality in the high HbA1c variability group was nearly 2·5 (HR: 2·38, 95% confidence interval [CI]: 1·99 − 2·84, K-means) and 4 (HR: 3·76, 95% CI: 3·06 − 4·64, K-means) times higher than that in the low HbA1c variability group, respectively (Table S6). The relationship between either the HVS or the SD and MACEs could be presented as a J-shaped curve, and this relationship did not change with the intensity of the treatment (Figure S2). The results of GAM showed that as HVS and SD increase, the heightened risk brought about by intensive glucose management compared to standard glucose management became higher (Figure S3). Regarding hypoglycemic events, as the degree of variability increased, the incidence of hypoglycemic events significantly raised (Table S6).

### HbA1c variability and intensive blood glucose treatment

**Fig. 1:**
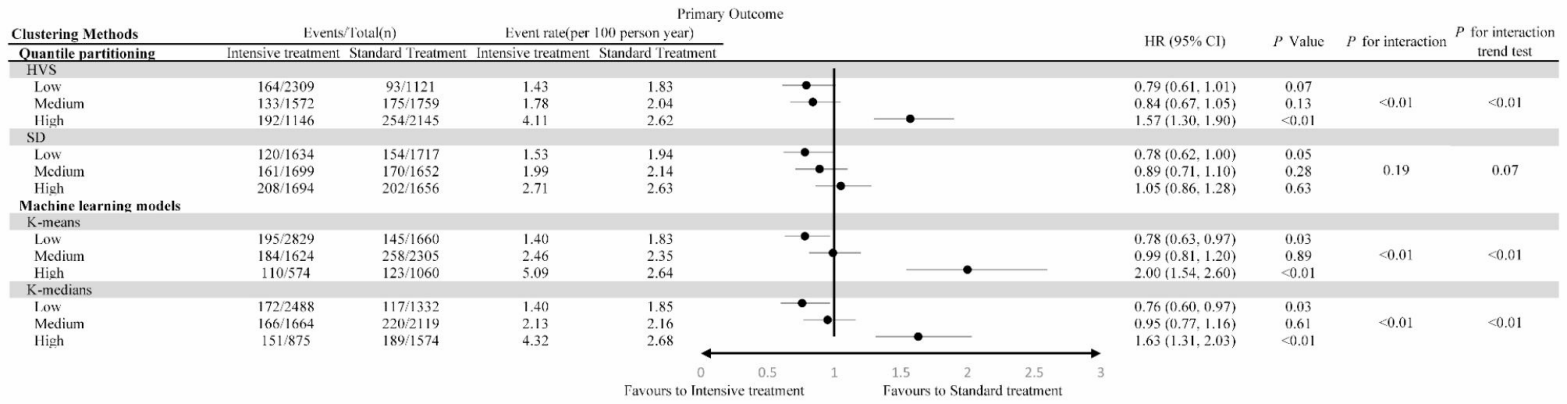
The primary outcome in participants randomized to the intensive compared with the standard blood glucose treatment group, stratified by HbA1c variability levels

Participants were divided into six groups according to HbA1c variability and intensive treatment. The group with high HbA1c variability and intensive treatment had the highest risk of MACEs, while that with low variability and intensive treatment had the lowest risk (Figure S4). There was a significant interaction between intensive treatment and HbA1c variability grouping (*P* < 0·01, K-means and K-medians). Additionally, intensive treatment significantly reduced the risk of MACEs in the low HbA1c variability group (HR: 0·78, 95% CI: 0·63 − 0·97, K-means) but significantly increased the risk in the high variability group (HR: 2·00, 95% CI: 1·54 − 2·60, K-means) (Fig. 1). In the low variability group, intensive treatment primarily reduced the risk of non-fatal MI (HR: 0·73, 95% CI: 0·56 − 0·97, K-means) and non-fatal stroke (HR: 0·61, 95% CI: 0·36-1.04, K-means) (Table S7).

**Fig. 2:**
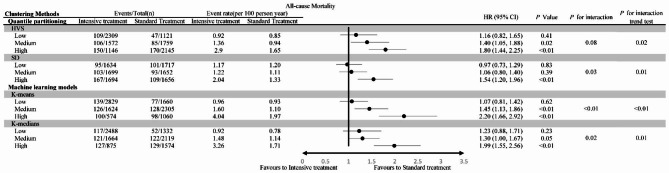
The all-cause mortality in participants randomized to the intensive compared with the standard blood glucose treatment group, stratified by HbA1c variability levels

For all-cause mortality, there was also a significant interaction between HbA1c variability and intensive treatment (*P* < 0·01, K-means and K-medians). Similarly, intensive treatment did not increase the mortality risk in the low HbA1c variability group (HR: 1·07, 95% CI: 0·81 − 1·42, K-means), but significantly increased the mortality risk in the high HbA1c variability group (HR: 2·20, 95% CI: 1·66 − 2·92, K-means) (Fig. 2).

Regardless of the categorization by degree of variability, intensive treatment significantly increased the incidence of hypoglycemic events. However, as variability increases, the magnitude of risk enhancement correspondingly decreases (*P* for interaction trend test = 0.02, both K-means and K-medians, Table S8). In the low variability group, intensive treatment did not increase the incidence of non-hypoglycemic related SAE (Serious Adverse Events). However, in the medium and high variability groups, there was a significant increase in the incidence of non-hypoglycemic related SAE. Furthermore, in the standard treatment group, an increase in variability did not elevate the incidence of non-hypoglycemic related SAE. Yet, in the intensive treatment group, an increase in variability significantly raised the incidence of non-hypoglycemic related SAE (Figure S5).

Whether considering the risk of MACEs, all-cause mortality or the hypoglycemic events, as the variability grouping increases, the benefits of intensive management disappeared and instead significantly increased the risk. There was a dose-response relationship between variability grouping and the risk of intensive management (*P* for interaction trend test < 0.05, both K-means and K-medians, MACEs, all-cause mortality and hypoglycemic events) (Figs. 1 and 2, Table S8). This indicated that in different variability groupings, the change in mean HbA1c brought about different changes in risk.

### The mean HbA1c value from all visits and the risk of outcomes in different k-means variability groups

**Fig. 3:**
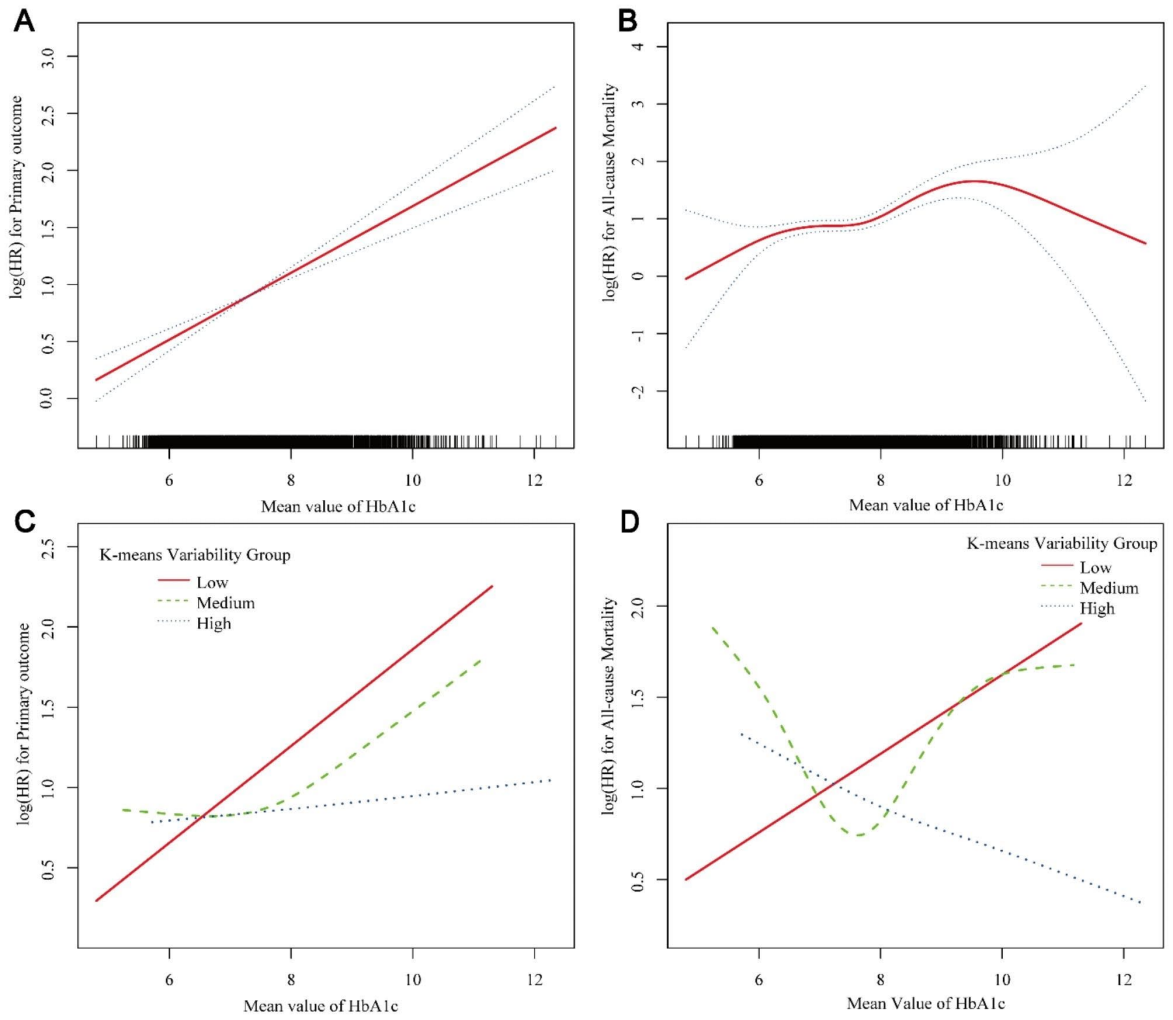
Association between the mean HbA1c value from all visits and the risk of primary outcome and all-cause mortality. **A** & **B**: Among all participants. **C** & **D**: Comparison among different HbA1c variability groups. The solid red line represents the estimated hazard ratio, while the dotted blue line indicates the 95% confidence interval

Given that intensive glucose-lowering strategies demonstrated varying effects across different variability groups, to further ascertain the role of HbA1c variability in influencing the therapeutic efficacy of glucose-lowering in patients, we computed the mean HbA1c value for each patient during the follow-up period. Additionally, GAM was employed to elucidate the relationship between the outcomes’ risk and the average HbA1c values. We observed that overall, as the mean HbA1c value increased, the risk of MACEs consistently escalated. However, the risk for all-cause mortality exhibited a more complex curve (Fig. 3A, B).

In different variability groups, the influence of average HbA1c on the risk of MACEs varied substantially (Fig. 3C, Figure S6). As illustrated in the GAM, in the low variability group, the risk of MACEs significantly increased with the rise of the mean HbA1c (HR: 1·33, 95% CI: 1·14 − 1·56, P < 0·01). Moreover, the log-likelihood ratio test did not support a curved relationship (*P* for log-likelihood ratio test = 0·46) (Table 2). However, in the medium variability group, the risk remained unchanged with changes in the mean HbA1c value until reaching an inflection point (HR: 0·88, 95% CI: 0·67 − 1·15, *P* = 0·36); beyond this point (7·49% [7·34%, 7·65%]), the risk of MACEs increased with the rise of the mean HbA1c (HR: 1·38, 95% CI: 1·11 − 1·71, *P* < 0·01). In the high variability group, the average HbA1c had no significant impact on the MACEs’ risk, irrespective of the model used. Furthermore, the variability grouping exhibited a significant interaction effect with the relationship between the average HbA1c and the MACEs’ risk (*P* for interaction < 0·05).

**Table 2:** Results of the two-piecewise linear-regression model of mean value of HbA1c and outcomes

**Primary Outcome**				
K-means Variability Group	Low Variability	Medium Variability	High Variability	Total
Mean Value of HbA1c				*P* for interaction: 0.05
One linear-regression model	1.33 (1.14, 1.56) *P* < 0.01	1.14 (0.99, 1.30) *P =* 0.06	1.06 (0.90, 1.23) *P =* 0.49	1.16 (1.07, 1.27) *P* < 0.01
Two-piecewise linear-regression model				
Inflection point (K and 95%CI) (%)	6.74 (6.57, 6.95)	7.49 (7.34, 7.65)	7.88 (7.68, 8.09)	8.82 (8.17, 8.87)
<K Effect size β (95%CI)	1.12 (0.70, 1.79) *P* = 0.63	0.88 (0.67, 1.15) *P* = 0.36	0.80 (0.54, 1.18) *P* = 0.27	1.18 (1.07, 1.31) *P* < 0.01
>K Effect size β (95%CI)	1.42 (1.13, 1.78) *P* < 0.01	1.38 (1.11, 1.71) *P* < 0.01	1.19 (0.96, 1.47) *P* = 0.11	1.08 (0.79, 1.47) *P* = 0.61
P for Log-likelihood ratio test	0.46	0.04	0.15	0.60
**All-cause Mortality**				
K-means Variability Group	Low Variability	Medium Variability	High Variability	Total
Mean Value of HbA1c				*P* for interaction: 0.02
One linear-regression model	1.23 (1.01, 1.51) *P* = 0.04	0.92 (0.76, 1.11) *P* = 0.38	0.86 (0.72, 1.03) *P =* 0.10	0.98 (0.88, 1.09) *P* = 0.65
Two-piecewise linear-regression model				
Inflection point (K and 95%CI) (%)	5.99 (5.97, 6.21)	7.44 (7.30, 7.59)	7.86 (7.66, 8.06)	6.11 (6.10, 6.43)
<K Effect size β (95%CI)	None	0.49 (0.36, 0.69) *P < 0.01*	0.63 (0.42, 0.95) *P < 0.01*	None
>K Effect size β (95%CI)	1.19 (0.96, 1.47) *P =* 0.11	1.57 (1.19, 2.08) *P* < 0.01	1.01 (0.78, 1.31) *P* = 0.11	0.95 (0.85, 1.07) *P* = 0.14
P for Log-likelihood ratio test	0.29	< 0.01	0.12	0.10

If the p-value of the log-likelihood ratio test is less than 0.05, it supports that the relationship between the dependent and independent variables is curvilinear rather than linear

The relationship between mean HbA1c and the risk of all-cause mortality varied across different variability groups (Fig. 3D, Figure S6). In the low variability group, the risk of all-cause mortality increased with the rise of the mean HbA1c (HR: 1·23, 95% CI: 1·01–1·51) *P* = 0·04), and the log-likelihood ratio test did not indicate a curvilinear relationship between them (*P* = 0·29). Intriguingly, within the medium variability group, the relationship between mean HbA1c and all-cause mortality risk exhibited a U-shaped curve (*P* for log-likelihood ratio test < 0·01). When the mean HbA1c was below the inflection point (7·44, 95%CI: 7·30 − 7·59), the mortality risk decreased with an increase in mean HbA1c (HR: 0·49, 95% CI: 0·36 − 0·69, *P* < 0·01). However, once the mean HbA1c surpassed this inflection point, its increase corresponded with a heightened mortality risk (HR: 1·57, 95% CI: 1·19 − 2·08, *P* < 0·01). Surprisingly, in the high variability cohort, when the mean HbA1c was below the inflection point (7·86, 95% CI: 7·66 − 8·06), there was a significant reduction in mortality risk with an increasing mean HbA1c (HR: 0·63, 95% CI: 0·42 − 0·95, *P* < 0·01). However, once the mean HbA1c exceeded this point, it no longer had a discernible impact on the mortality risk (HR: 1·01, 95% CI: 0·78 − 1·31, *P* = 0·11). A significant interaction was observed between variability grouping and the relationship between the mean HbA1c and the risk of all-cause mortality (*P* for interaction = 0.02). We also present the analysis for hypoglycemic events in Table S9.

### Sensitivity analysis

Sensitivity analysis showed that our results remained robust after the exclusion of the MACEs that occurred in the first year of follow-up. HbA1c variability still interacted significantly with intensive treatment, and intensive treatment significantly reduced the risk of MACEs (HR: 0·74, 95% CI: 0·58 − 0·94, K-means) and did not increase the mortality risk (HR: 1·14, 95% CI: 0·84 − 1·54, K-means) in the low variability group (Table S10). After excluding these patients, the relationship between mean HbA1c and both MACE and all-cause mortality risk remained unchanged across the different variability groups (Table S11). To further validate the robustness of our results, we employed VIM as a substitute for SD. VIM was utilized to assess the variability of HbA1c in another secondary analysis of ACCORD study [20]. After clustering HVS and VIM using the K-means method, the findings, ranging from group sizes to the impact on risk of adverse outcomes, closely aligned with our main results (Table S12, S13).

## Discussion

In this *post-hoc* analysis, we found that HbA1c variability can significantly affect the efficacy of glucose-lowering treatments. Within different variability groups, intensive glucose-lowering treatment yielded varying outcomes. Specifically, in the low HbA1c variability group, intensive glucose lowering significantly decreased the risk of MACEs without increasing the risk of all-cause mortality. Conversely, in the high variability group, intensive glucose lowering significantly heightened the risk for both MACEs and all-cause mortality. Additionally, the optimal target for long-term mean HbA1c varied among different variability groups. Our findings potentially offer clinical implications in guiding patients with T2DM to determine their ideal glucose-lowering target.

Numerous studies have shown that in patients with T2DM, lower levels of HbA1c were associated with lower MACEs risks [2, 21–23]. Our data also indicated that in the overall participants of the ACCORD study, the risk of MACEs increased with the rise of long-term mean HbA1c levels, and the risk of all-cause mortality was not related to long-term mean HbA1c levels. However, intensive blood glucose management, compared to standard blood glucose management, not only did not reduce the risk of MACEs but also increased the risk of all-cause mortality [4]. This result was puzzling, suggesting that there could be some factors that have not received enough attention previously that affected the intensive management. Current research has already established that glycemic variability is an independent predictor of adverse outcomes in patients with T2DM [24, 25]. In recent years, the variability of HbA1c has gradually gained attention, furthermore, many studies have demonstrated a significant association between the variability of HbA1c and adverse outcomes [20, 26]. In 2018, a new index for measuring variability, HVS, has been proposed. Previous studies have reported a significant correlation between the HVS and adverse outcomes [27, 28]. In these studies, using the HVS had many advantages over the SD and the CV, but still had some disadvantages. HVS is more translatable in clinical practice (as it can be interpreted as the percentage of total HbA1c measures that vary by 0·5% or 5·5 mmol/mol) [29]. Although the HVS can show the frequency of HbA1c variability well, it still ignores the magnitude of HbA1c variability. Our research combines HVS with SD and systematically demonstrates the impact of HbA1c variability.

Previous studies aimed at evaluating the effect of intensive blood glucose treatment did not reach a consistent conclusion, which may be due to HbA1c variability. Of note, a secondary analysis using data from the Veterans Affairs Diabetes Trial (VADT) showed that blood glucose variability was only associated with adverse events in the intensive treatment group, suggesting that excessive blood glucose variability may neutralize the benefits of lower blood glucose levels [30, 31]. However, our study found that higher variability in HbA1c brings higher risks regardless of treatment strategies. Our results found that in patients with low HbA1c variability, intensive treatment reduced the risk of cardiovascular events by 22% without increasing the risk of all-cause mortality; for these patients, whether considering the primary outcome or the risk of all-cause mortality, a lower mean HbA1c corresponded to a reduced risk. This is consistent with the conclusion of previous studies that patients with low and stable blood glucose levels have the lowest risk [27]. Our research findings further emphasize the importance of HbA1c variability in the T2DM management, showing that long-term variability could serve as a factor guiding the glycemic control treatment in patients with T2DM. In patients with high HbA1c variability, intensive treatment not only failed to bring benefits but also significantly increased the risk of cardiovascular events and all-cause mortality, our results indicate that the altered risk of hypoglycemic events was insufficient to account for the increased adverse event risk observed in this subset of patients. This might be attributable to the significant rise in the incidence of non-hypoglycemic related SAE in the intensive treatment group. In these patients, maintaining a higher HbA1c level results in a lower risk. The level of mean HbA1c did not influence the risk of the MACEs. However, based on our results, patients with a mean HbA1c of around 7·88% had the lowest risk of all-cause mortality. A mean HbA1c below this value could lead to an increased risk, while values above this threshold did not impact the risk. These results suggested that in patients with T2DM, low levels of HbA1c should not be pursued excessively; instead, HbA1c should be controlled to maintain stability at the ideal level. However, due to the emergence of novel antidiabetic drugs such as Sodium-Glucose Co-Transporter 2 inhibitors (SGLT2i) and Glucagon-Like Peptide-1 Receptor Agonists (GLP-1RA), the current pharmacological treatment for T2DM has many differences from that used in the ACCORD study. Therefore, this result still requires further up-to-date research for confirmation.

Prior research has indicated that patients with high HbA1c variability presented with more cardiovascular risk factors at baseline [32]. Therefore, the association between HbA1c variability and the risk of adverse events may not be a feature of HbA1c variability itself, but a sign of baseline differences in the patients’ characteristics. In this study, the association between HbA1c variability and the outcome remained robust even after adjustment for major cardiovascular risk factors, although some residual unadjusted risks may remain. The American Diabetes Association guidelines and American Heart Association scientific statement recommend individualization of HbA1c targets using a patient-centered approach: <7% (53 mmol/mol) for most nonpregnant adults; <6·5% for young patients with a long life expectancy and no significant cardiovascular disease; and less stringent targets (i.e., < 8%) for those with a history of severe hypoglycemia, limited life expectancy, and advanced microvascular or macrovascular complications [33, 34]. Our findings suggested that HbA1c variability could be one of the risk factors guiding patients’ glucose lowering targets, and thus expanded the population that could benefit from intensive glucose lowering strategies. Our results showed that patients with high HbA1c variability had poor outcomes with intensive treatment for blood glucose control compared with other patients, and they also had greater HbA1c variability than the standard treatment group. According to our findings, a potential approach for patients with T2DM exhibiting long-term high HbA1c variability was to administer cautious glucose-lowering treatments. If the glucose-lowering effects were not significant, the strategy should be shifted towards stabilizing blood glucose levels. This could potentially prevent the increase in cardiovascular risks associated with aggressive glucose reduction. For patients with low HbA1c variability, the lower the mean HbA1c, the lower the risk. For those with moderate HbA1c variability, patients with a mean HbA1c level around 7·5% have the lowest risk. Meanwhile, for patients with high HbA1c variability, mean HbA1c does not influence the risk of adverse cardiovascular events, but a value around 7.8% is associated with the lowest all-cause mortality rate. Our results suggest that the long-term HbA1c variability of patients can guide the optimal blood glucose control target. How to establish a more effective risk grading criteria may be the future research direction for the treatment of T2DM.

Our study has many strengths. First, we systematically expounded the relationship between blood glucose-lowering treatment strategies and HbA1c variability for the first time. Second, our study benefitted from a large sample size and long follow-up period. Third, we used machine learning algorithms to classify the population into different HbA1c variability groups. However, our study has some limitations. First, the ACCORD study was not designed to evaluate the relationship between blood glucose-lowering treatment strategies and HbA1c variability; thus, the *post-hoc* analysis had its inherent limitations and could not lead to causal inferences. Additionally, the participants included in the ACCORD study did not represent the general population; thus, more general population studies are needed to verify our conclusions. Over the past decade, the therapeutic strategies for T2DM have undergone significant advancements. The treatment approach adopted in the ACCORD study is increasingly being supplanted by emerging medications, such as SGLT2i and GLP-1RA. Therefore, caution is warranted when interpreting and applying the findings of this study in clinical practice. Finally, as the high variability group had higher rates of smoking, more heart disease, higher BMI, higher cholesterol, and many other factors, there may be unobserved confounders such as exercise and diet to consider.

## Conclusion

In conclusion, our findings suggest that long-term HbA1c variability can guide the long-term glycemic control targets for patients with T2DM. For those with low HbA1c variability, the lower the mean HbA1c, the lower the risk, and intensive glucose management can yield better outcomes. In patients with medium HbA1c variability, intensive glycemic control does not reduce the risk of cardiovascular events or all-cause mortality and a mean HbA1c level around 7·5% is associated with the lowest risk. Conversely, for those with high HbA1c variability, mean HbA1c does not influence the risk of MACEs. However, a value around 7·8% corresponds to the lowest all-cause mortality risk. For this group, having an excessively high value does not increase the all-cause mortality risk, but an excessively low value does elevate it. Further studies, especially those with samples that reflect the general population and research in the real-world setting of patients with T2DM undergoing the latest therapeutic approaches, are essential to validate the conclusions of this study.

### Electronic supplementary material

Below is the link to the electronic supplementary material.

Supplementary Material 1

## Data Availability

Data are available from the Biologic Specimen and Data Repository Information Coordinating Center (BioLINCC).
