# Supplementary material for: Glycemic control, HbA1c variability, and major cardiovascular adverse outcomes in type 2 diabetes patients with elevated cardiovascular risk: insights from the ACCORD study
Source: Cardiovasc Diabetol. 2023 Oct 27;22:287. doi: 10.1186/s12933-023-02026-9 (PMC10612188; doi:10.1186/s12933-023-02026-9)
Supplement: Supplementary file 1 — Supplementary Material 1 [file 12933_2023_2026_MOESM1_ESM.pdf]

Table S1. Internal Validation Metrics of different categories numbers.

Clustering Methods	K-means		K-medians	
Categories numbers	Davies-Bouldin Index	Silhouette Index	Davies-Bouldin Index	Silhouette Index
2	0.59	0.56	0.60	0.55
3	0.57	0.52	0.57	0.52
4	0.57	0.50	0.57	0.49
5	2.02	0.38	0.57	0.47
6	3.40	0.38	1.08	0.37
7	1.40	0.38	0.85	0.33
8	1.43	0.23	1.27	0.30
9	2.25	0.26	0.95	0.27
10	1.49	0.25	3.72	0.31

Low values of the Davies-Bouldin Index and high values of the Silhouette Indexes reflected better clustering with less similarity, so the clusters of data show different characteristics.

Table S2. Baseline characteristics and crude endpoints of the study participants stratified by variability and treatment strategy.

K-means Variability Group	Low		Medium		High		Total	
	Standard arm	Intensive arm	Standard arm	Intensive arm	Standard arm	Intensive arm	Standard arm	Intensive arm
N	1660	2829	2305	1624	1060	574	5025	5027
Age	64.1±6.7	63.3±6.5*	62.4±6.4	62.3±6.7	61.5±6.7	61.4±6.6	62.7±6.6	62.7±6.6
Female	639(38.5%)	1058(37.4%)	864(37.5%)	635(39.1%)	417(39.3%)	243(42.3%)	1920(38.2%)	1936(38.5%)
Race or ethnic group (%)								
White	1116(67.2%)	1886(66.7%)	1469(63.7%)	948(58.4%)*	562(53.0%)	300(52.3%)	3147(62.6%)	3134(62.3%)
Black	235(14.2%)	433(15.3%)	412(17.9%)	369(22.7%)	280(26.4%)	175(30.5%)	927(18.4%)	977(19.4%)
Hispanic	89(5.4%)	161(5.7%)	159(6.9%)	137(8.4%)	121(11.4%)	52(9.1%)	369(7.3%)	350(7.0%)
Other	220(13.3$)	349(12.3%)	265(5.6%)	170(10.5%)	97(9.2%)	47(8.2%)	582(11.6%	566(11.3%)
CVD History (%)	528(31.8%)	918(32.4%)	804(34.9%)	628(38.7%)*	402(37.9%)	236(41.1%)	1734(34.5%)	1782(35.4%)
Education (%)								
Less than high school	191(11.5%)	385(18.6%)	322(14.0%)	280(17.3%)*	172(16.3%)	114(19.9%)	685(13.6%)	779(15.5%)
High-school graduate	425(25.6%)	755(26.7%)	616(26.7%)	422(26.0%)	303(28.5%)	142(24.7%)	1343(26.7%)	1319(26.3%)
Some college	549(33.1%)	917(32.4%)	766(33.2%)	536(33.0%)	343(32.4%)	187(32.6%)	1658(33.0%)	1640(32.6%)
College degree or higher	494(29.8%)	770(27.2%)	601(26.1%)	385(23.7%)	241(22.8%)	131(22.8%)	1336(26.6%)	1286(25.6%)
Baseline HbA$_{1C}$ (%)	8.0 ± 0.8	8.0 ± 0.9	8.4 ± 1.0	8.6 ± 1.1*	8.7 ± 1.2	8.9 ± 1.2*	8.3 ± 1.1	8.3 ± 1.1
Blood pressure (mm Hg)								
Systolic	136.3±16.7	135.5±16.1	136.3±17.1	136.8±17.8	136.8±18.1	137.9±18.3	136.4±17.2	136.2±17.0
Diastolic	74.2±10.3	74.5±10.2	75.0±10.6	74.8±11.0	76.0±11.2	76.2±11.4	75.0±10.7	74.8±10.6
Heart rate(BPM)	71.6±11.6	72.2±11.5	72.6±11.63	73.0±11.8	73.8±12.0	74.7±12.4	72.6±11.7	72.7±11.7
Body-mass index	31.4±5.1	32.0±5.3*	32.6±5.4	32.5±5.5	32.7±5.6	32.9±5.6	32.2±5.4	32.2±5.4
Cholesterol (mg/dl)								
Total	179.7±38.7	181.8±40.3	185.0±42.6	184.7±43.7	185.5±43.6	187.5±44.9	183.3±41.6	183.4±42.0
Low-density lipoprotein	102.9±32.1	103.9±32.9	105.7±34.2	105.5±34.2	106.3±35.3	107.8±37.7	104.9±33.8	104.9±33.9
High-density lipoprotein	43.0±12.0	41.8±11.4*	41.3±11.0	41.8±12.0	41.4±11.5	42.3±13.2	41.9±11.5	41.8±11.8
Triglyceride (mg/dl)	173.5±111.8	187.2±132.5*	197.1±152.9	194.3±162.9	199.6±185.7	200.1±179.0	189.8±149.1	190.9±148.7
Fasting serum glucose (mg/dl)	165.2±46.7	168.6±48.6*	177.8±57.0	181.1±60.0	188.0±65.8	187.1±71.6	175.8±56.5	174.8±55.9
Estimated GFR, mL *min^{-1}*1.73 m^{-2}	90.3±23.4	90.6±25	91.7±32.8	90.7±26.3	92.2±25.4	92.1±27.4	91.4±28.5	90.8±25.7
History physical exam (%)								
Protein in urine	295(17.8%)	493(17.4%)	476(20.7%)	357(22.0%)	231(21.8%)	142(24.7%)	1002(19.9%)	992(19.7%)
Heart failure	66(4.0%)	107(3.8%)	102(4.4%)	100(6.2%)*	71(6.7%)	28(4.9%)	239(4.8%)	235(4.7%)
Neuropathy	423(25.5%)	670(23.7%)	657(28.5%)	442(27.2%)	305(28.8%)	180(31.4%)	1385(27.6%)	1292(25.7%)*
Depression	323(19.5%)	570(20.2%)	569(24.7%)	426(26.2%)	310(29.2%)	172(30.0%)	1202(23.9%)	1168(23.2%)
Eye disease	536(32.3%)	831(29.4%)*	731(31.7%)	535(32.9%)	337(31.8%)	169(29.4%)	1604(31.9%)	1535(30.5%)
Smoked cigarettes in last 30 days	182(11.0%)	357(12.6%)	315(13.7%)	249(15.3%)	190(17.9%)	99(17.2%)	687(13.7%)	705(14.0%)
Crude outcomes (%)								
Primary outcome	145(8.7%)	195(6.9%)*	258(11.2%)	184(11.3%)	123(11.6%)	110(19.2%)*	526(10.5%)	489(9.7%)
All-cause mortality	77(4.6%)	139(4.9%)	128(5.6%)	126(7.8%)*	98(9.2%)	100(17.4%)*	303(6.0%)	365(7.3%)*
CVD-mortality	30(1.8%)	63(2.2%)	56(2.4%)	62(3.8%)*	45(4.2%)	50(8.7%)*	131(2.6%)	175(3.52%)*
Non-fatal MI	94(5.7%)	119(4.2%)*	179(7.8%)	114(7.0%)	66(6.2%)	52(9.1%)*	339(6.7%)	285(5.7%)*
Non-fatal Stroke	28(1.7%)	28(1.0%)*	39(1.7%)	29(1.8%)	26(2.5%)	43(4.0%)	93(1.9%)	80(1.6%)

Total Stroke	31(1.9%)	32(1.1%)*	44(1.9%)	33(2.0%)	28(2.6%)	24(4.2%)	103(2.0%)	89(1.8%)

*, Statistically significant compared to the standard management approach, P<0.05. CVD, cardiovascular diseases; HbA1c, Hemoglobin A1c; HVS, HbA1c variability score; GFR, glomerular filtration rate; MI, myocardial infarction. Plus–minus values are means ± SD. Race and ethnic group were self-reported. The body mass index is the weight in kilograms divided by the square of the height in meters.

Table S3. The cut-off value of classification of HbA1c variability

Clustering Methods	Number in Group (%)		
Quantile partitioning	Low Variability	Medium Variability	High Variability
HVS	3430(34.1%)	3331(33.1%)	3291(32.8%)
SD	3351(33.3%)	3351(33.3%)	3350(33.3%)
Machine learning models			
K-means	4489(44.7%)	3929(39.1%)	1634(16.3%)
K-medians	3820(38%)	3783(37.6%)	2449(24.4%)
		Mean HVS	
Quantile partitioning	Low Variability	Medium Variability	High Variability
HVS	15.87 ± 7.25	36.58 ± 5.78	64.70 ± 14.78
SD	25.12 ± 17.66	38.87 ± 19.03	52.17 ± 21.54
Machine learning models			
K-means	19.29 ± 8.92	45.44 ± 8.26	75.95 ± 13.01
K-medians	17.19 ± 7.92	40.63 ± 7.45	69.35 ± 14.38
		Mean SD	
Quantile partitioning	Low Variability	Medium Variability	High Variability
HVS	0.53 ± 0.23	0.69 ± 0.26	0.92 ± 0.39
SD	0.41 ± 0.09	0.64 ± 0.07	1.08 ± 0.32
Machine learning models			
K-means	0.53 ± 0.21	0.77 ± 0.28	1.06 ± 0.42
K-medians	0.52 ± 0.22	0.70 ± 0.24	1.02 ± 0.39

Table S4. Classification of HbA1c variability

Clustering Methods	Baseline HbA1c (%)					
	Low Variability		Medium Variability		High Variability	
Quantile partitioning	Intensive treatment	Standard Treatment	Intensive treatment	Standard Treatment	Intensive treatment	Standard Treatment
HVS	8.03 ± 0.94*	7.91 ± 0.83	8.36 ± 1.04*	8.25 ± 0.98	8.73 ± 1.14*	8.57 ± 1.14
SD	7.55 ± 0.57*	7.79 ± 0.63	8.15 ± 0.67*	8.24 ± 0.83	9.15 ± 1.13*	8.92 ± 1.27
Machine learning models						
K-means	8.01 ± 0.92*	7.95 ± 0.82	8.57 ± 1.07*	8.37 ± 1.03	8.91 ± 1.19*	8.74 ± 1.22
K-medians	8.00 ± 0.92*	7.91 ± 0.80	8.40 ± 1.03*	8.28 ± 0.97	8.92 ± 1.17*	8.69 ± 1.20
	Mean HbA_{1c} (%)					
	Low Variability		Medium Variability		High Variability	
Quantile partitioning	Intensive treatment	Standard Treatment	Intensive treatment	Standard Treatment	Intensive treatment	Standard Treatment
HVS	6.55 ± 0.50*	7.40 ± 0.52	6.93 ± 0.59*	7.65 ± 0.58	7.61 ± 0.84*	8.06 ± 0.78
SD	6.68 ± 0.59*	7.50 ± 0.52	6.78 ± 0.63	7.70 ± 0.59	7.26 ± 0.86	8.11 ± 0.84
Machine learning models						
K-means	6.59 ± 0.52*	7.45 ± 0.53	7.13 ± 0.66*	7.75 ± 0.61	7.86 ± 0.87*	8.28 ± 0.85
K-medians	6.56 ± 0.51*	7.42 ± 0.51	6.99 ± 0.61*	7.69 ± 0.59	7.73 ± 0.85*	8.16 ± 0.81
	HVS					
	Low Variability		Medium Variability		High Variability	
Quantile partitioning	Intensive treatment	Standard Treatment	Intensive treatment	Standard Treatment	Intensive treatment	Standard Treatment
HVS	15.76 ± 6.80	16.10 ± 8.09	35.78 ± 5.76*	37.30 ± 5.71	65.19 ± 16.33	64.44 ± 13.87
SD	21.74 ± 16.83*	28.34 ± 17.83	31.97 ± 18.14*	45.97 ± 17.22	45.76 ± 22.33*	58.74 ± 18.54
Machine learning models						
K-means	18.36 ± 8.29*	20.88 ± 9.68	43.65 ± 8.06*	46.69 ± 8.18	77.54 ± 14.50*	75.08 ± 12.04
K-medians	16.68 ± 7.36*	18.14 ± 8.81	38.99 ± 7.26*	41.92 ± 7.34	69.66 ± 16.21	69.17 ± 13.26
	SD					
	Low Variability		Medium Variability		High Variability	
Quantile partitioning	Intensive treatment	Standard Treatment	Intensive treatment	Standard Treatment	Intensive treatment	Standard Treatment
HVS	0.57 ± 0.23*	0.44 ± 0.19	0.74 ± 0.28*	0.64 ± 0.24	0.97 ± 0.42*	0.89 ± 0.36
SD	0.42 ± 0.09*	0.40 ± 0.09	0.64 ± 0.07	0.64 ± 0.07	1.08 ± 0.32	1.08 ± 0.31
Machine learning models						
K-means	0.58 ± 0.22*	0.46 ± 0.18	0.83 ± 0.31*	0.72 ± 0.25	1.09 ± 0.46*	1.04 ± 0.40
K-medians	0.57 ± 0.22*	0.44 ± 0.18	0.75 ± 0.26*	0.66 ± 0.22	1.07 ± 0.43*	0.99 ± 0.37

*The difference was statistically significant compared to the standard treatment group.

Table S5. Comparison of Internal Validation Metrics.

Performance Index	Calinski-Harabaz Index	Davies-Bouldin Index	Silhouette Index
Clustering Methods			
Quantile partitioning			
HVS	20024.318	0.577	0.506
SD	1617.038	2.313	0.002
Machine learning models			
K-means	23041.551	0.570	0.523
K-medians	21451.039	0.597	0.499

Low values of the Davies-Bouldin Index and high values of the Calinski-Harabaz and Silhouette Indexes reflected better clustering with less similarity, so the clusters of data show different characteristics.

Table S6. Comparing Risk of Different Outcomes Among Participants with Different levels of HbA1c variability.

HbA_{1c} Variability Levels	Medium vs Low		High vs Low	
	HR (95% CI)	*P* Value	HR (95% CI)	*P* Value
Primary outcome				
Quantile partitioning				
HVS	1.21 (1.02, 1.43)	0.03	2.12 (1.79, 2.50)	<0.01
SD	1.16 (0.99, 1.36)	0.08	1.56 (1.33, 1.82)	<0.01
Machine learning models				
K-means	1.54 (1.33, 1.79)	<0.01	2.38 (1.99, 2.84)	<0.01
K-medians	1.37 (1.17, 1.60)	<0.01	2.23 (1.88, 2.65)	<0.01
All-cause mortality				
Quantile partitioning				
HVS	1.35 (1.09, 1.68)	<0.01	3.01 (2.45, 3.70)	<0.01
SD	0.96 (0.79, 1.18)	0.72	1.52 (1.26, 1.84)	<0.01
Machine learning models				
K-means	1.55 (1.28, 1.87)	<0.01	3.76 (3.06, 4.64)	<0.01
K-medians	1.61 (1.31, 1.96)	<0.01	3.38 (2.75, 4.17)	<0.01
Hypoglycemic events				
Quantile partitioning				
HVS	1.32(1.14, 1.52)	<0.01	1.78(1.53, 2.06)	<0.01
SD	1.26(1.09, 1.46)	<0.01	1.55(1.34, 1.79)	<0.01
Machine learning models				
K-means	1.46(1.29, 1.66)	<0.01	1.88(1.59, 2.23)	<0.01
K-medians	1.40(1.23, 1.61)	<0.01	1.87(1.61, 2.18)	<0.01

Table S7. The HRs for outcomes in participants randomized to the intensive compared with the standard blood glucose treatment group, stratified by HbA1c variability levels.

K-means Variability Group	HR (95% CI) for Intensive Treatment	*P* Value	*P* for interaction
		**CVD death**	
Low	1.23(0.78,1.91)	0.36	
Medium	1.55(1.08,2.23)	0.02	0.04
High	2.48(1.64,3.75)	<0.01	
		**Non-Fatal MI**	
Low	0.73(0.56,0.97)	0.03	
Medium	0.88(0.69,1.11)	0.29	<0.01
High	1.71(1.19,2.47)	<0.01	
		**Non-Fatal Stroke**	
Low	0.63(0.38,1.03)	0.07	
Medium	1.11(0.70,1.76)	0.66	0.03
High	1.70(0.97,2.96)	0.06	

CVD death, mortality caused by cardiovascular reasons. MI, myocardial infarction

Table S8. The Hypoglycemia events in participants randomized to the intensive compared with the standard blood glucose treatment group, stratified by HbA1c variability levels.

	Hypoglycemic events							
	Events/Total(n)		Event rate(per 100 person year)					
**Clustering Methods**	Intensive treatment	Standard Treatment	Intensive treatment	Standard Treatment	HR (95% CI) for Intensive Treatment	*P* Value	*P* for interaction	*P* for interaction trend test
**Quantile partitioning**								
HVS								
Low	350	38	3.35	0.71	4.92 (3.52, 6.89)	<0.01		
Medium	304	115	4.56	1.33	3.30 (2.66, 4.10)	<0.01	0.02	<0.01
High	241	183	5.97	1.92	2.88 (2.36, 3.50)	<0.01		
SD								
Low	254	82	3.64	1.03	3.50 (2.73, 4.50)	<0.01		
Medium	314	111	4.30	1.40	3.04 (2.44, 3.78)	<0.01	0.14	0.05
High	327	143	4.76	1.88	2.56 (2.10, 3.12)	<0.01		
**Machine learning models**								
K-means								
Low	439	69	3.46	0.86	4.12(3.19, 5.31)	<0.01		
Medium	337	168	5.10	1.53	3.20(2.65, 3.86)	<0.01	0.06	0.02
High	119	99	6.39	2.19	2.69(2.05, 3.54)	<0.01		
K-medians								
Low	379	47	3.38	0.74	4.78(3.53, 6.48)	<0.01		
Medium	328	152	4.75	1.50	3.03(2.49, 3.68)	<0.01	0.01	0.02
High	188	137	6.21	1.98	2.99(2.38, 3.74)	<0.01		

Table S9. Results of two-piecewise linear regression for hypoglycemic events.

	Hypoglycemic events			
K-means Variability Group	Low Variability	Medium Variability	High Variability	Total
Mean Value of HbA1c				*P* for interaction<0.01
One linear-regression model	0.72 (0.63, 0.83) *P*<0.01	0.93 (0.82, 1.06) *P*=0.30	1.01 (0.86,1.20) *P*=0.88	0.86 (0.80, 0.94) *P*<0.01
Two-piecewise linear-regression model				
Inflection point (K) (%)	6.42	7.69	8.45	7.79
<K Effect size β (95%CI)	1.86 (1.00, 3.47) *P*=0.05	0.53 (0.44, 0.65) *P*<0.01	0.69 (0.52, 0.91) *P*=0.01	0.63 (0.57, 1.71) *P*<0.01
>K Effect size β (95%CI)	0.59 (0.48, 0.71) *P*<0.01	1.92 (1.56, 2.36) *P*<0.01	1.54 (1.17, 2.04) *P*<0.01	1.42 (1.24, 1.64) *P*<0.01
Log-likelihood ratio test	<0.01	<0.01	<0.01	<0.01

Table S10. Sensitive analysis by excluding participants who had a follow-up time of fewer than 1 year.

	Primary Outcome			All-cause Mortality		
Clustering Methods	HR (95% CI) for Intensive Treatment	*P* Value	*P* for interaction	HR (95% CI) for Intensive Treatment	*P* Value	*P* for interaction
Quantile partitioning						
HVS			<0.01			0.22
Low	0.76 (0.57, 1.00)	0.05		1.29 (0.88, 1.89)	0.19	
Medium	0.84 (0.66, 1.07)	0.16		1.39 (1.03, 1.87)	0.03	
High	1.48 (1.19, 1.83)	<0.01		1.73 (1.35, 2.23)	<0.01	
SD			0.48			0.12
Low	0.79 (0.60, 1.02)	0.08		0.99 (0.72, 1.35)	0.93	
Medium	0.83 (0.66, 1.05)	0.13		1.09 (0.81, 1.47)	0.57	
High	0.98 (0.79, 1.22)	0.87		1.47 (1.13, 1.90)	<0.01	
Machine learning models						
K-means			<0.01			<0.01
Low	0.74 (0.58, 0.94)	0.01		1.14 (0.84, 1.54)	0.40	
Medium	0.98 (0.80, 1.21)	0.87		1.44 (1.11, 1.88)	<0.01	
High	1.94 (1.44, 2.60)	<0.01		2.23 (1.62, 3.08)	<0.01	
K-medians			<0.01			0.09
Low	0.74 (0.57, 0.96)	0.02		1.38 (0.96, 1.98)	0.08	
Medium	0.91 (0.73, 1.14)	0.41		1.29 (0.99, 1.69)	0.06	
High	1.60 (1.25, 2.05)	<0.01		1.95 (1.47, 2.58)	<0.01	

Table S11. Sensitive analysis of two-piecewise linear regression by excluding participants who had a follow-up time of fewer than 1 year.

**Primary Outcome**				
K-means Variability Group	Low Variability	Medium Variability	High Variability	Total
Mean Value of HbA1c				*P* for interaction: 0.02
One linear-regression model	1.37(1.16,1.63) *P*<0.01	1.11(0.96,1.29) *P*=0.16	1.03(0.86,1.23) *P*=0.73	1.16(1.06,1.28) *P*<0.01
Two-piecewise linear-regression model				
Inflection point (K) (%)	6.54	6.70	9.35	8.79
<K Effect size β (95%CI)	1.06(0.53,2.11) *P*=0.87	0.43(0.20,0.92) *P*=0.03	1.08(0.87,1.35) *P*=0.47	1.21(1.08,1.36) *P*<0.01
>K Effect size β (95%CI)	1.45(1.16,1.81) *P*<0.01	1.23(1.04,1.46) *P*<0.01	0.769(0.33,1.78) *P*=0.53	0.89(0.61,1.32) *P*=0.57
Log-likelihood ratio test	0.45	0.02	0.45	0.15
**All-cause Mortality**				
K-means Variability Group	Low Variability	Medium Variability	High Variability	Total
Mean Value of HbA1c				*P* for interaction <0.01
One linear-regression model	1.12(0.90,1.39) *P*=0.31	0.85(0.69,1.03) *P*=0.10	0.89(0.72,1.09) *P*=0.24	0.98(0.88,1.09) *P*=0.65
Two-piecewise linear-regression model				
Inflection point (K and 95%CI) (%)	5.99	7.43	7.24	6.11(6.10,6.43)
<K Effect size β (95%CI)	None	0.46(0.33,0.65) *P*<0.01	0.57(0.23,1.41) *P*=0.22	None
>K Effect size β (95%CI)	1.08(0.86,1.36) *P*=0.52	1.51(1.10,2.07) *P*<0.01	0.94(0.74,1.19) *P*=0.60	0.95(0.85,1.07) *P*=0.14
Log-likelihood ratio test	0.31	<0.01	0.35	0.10

Table S12. Sensitive analysis by clustering by HVS and VIM.

K-means Variability Group (Clustering by HVS and VIM)	Events/Total(n)		HR (95% CI) for Intensive Treatment	*P* Value	*P* for interaction	*P* for interaction trend test
	Intensive arm	Standard arm				
Primary outcomes						
Low	190/2742	145/1600	0.75 (0.60, 0.94)	0.01		
Medium	172/1647	247/2221	0.94 (0.77, 1.14)	0.51	<0.01	<0.01
High	102/550	121/1126	2.01 (1.54, 2.63)	<0.01		
All-cause Mortality						
Low	138/2742	72/1600	1.14 (0.85, 1.52)	0.39		
Medium	115/1647	121/2221	1.32 (1.02, 1.70)	0.04	<0.01	<0.01
High	83/550	91/1126	2.23 (1.64, 3.02)	<0.01		

VIM, variation independent of the mean.

Table S13. Sensitive analysis of two-piecewise linear regression by clustering by HVS and VIM.

K-means Variability Group (clustering by HVS and VIM)	**Primary Outcome** Low Variability	Medium Variability	High Variability	Total
Mean Value of HbA1c				*P* for interaction: 0.07
One linear-regression model	1.34 (1.15, 1.57) *P*<0.01	1.20 (1.05,1.37) *P*=0.01	1.08(0.92,1.26) *P*=0.37	1.20 (1.10, 1.31) *P*<0.01
Two-piecewise linear-regression model				
Inflection point (K) (%)	6.74	7.49	7.85	8.79
<K Effect size β (95%CI)	1.13 (0.70, 1.83) *P*=0.61	1.03 (0.79, 1.34) *P*=0.85	0.78 (0.54,1.14) *P*=0.20	1.22 (1.10,1.35) *P*<0.01
>K Effect size β (95%CI)	1.43 (1.14, 1.80) *P*<0.01	1.35 (1.09,1.66) *P*<0.01	1.25 (1.00, 1.56) *P*=0.05	1.10 (0.80, 1.51) *P*=0.56
Log-likelihood ratio test	0.47	0.2	0.08	0.56
K-means Variability Group (clustering by HVS and VIM)	**All-cause Mortality** Low Variability	Medium Variability	High Variability	Total
Mean Value of HbA1c				*P* for interaction :0.09
One linear-regression model	1.18 (0.96, 1.44) *P*=0.12	0.98 (0.82, 1.19) *P*=0.87	0.89 (0.73, 1.08) *P*=0.23	1.00 (0.90, 1.12) *P*=0.98
Two-piecewise linear-regression model				
Inflection point (K and 95%CI) (%)	5.99	7.56	7.25	6.12
<K Effect size β (95%CI)	None	0.65 (0.48, 0.88) *P*<0.01	0.42 (0.21, 0.85) *P*=0.01	None
>K Effect size β (95%CI)	1.14 (0.91, 1.41) *P*=0.25	1.54 (1.13, 2.10) *P*<0.01	1.02 (0.81, 1.27) *P*=0.89	0.98 (0.87, 1.10) *P*=0.69
Log-likelihood ratio test	0.33	<0.01	0.04	0.12

VIM, variation independent of the mean.

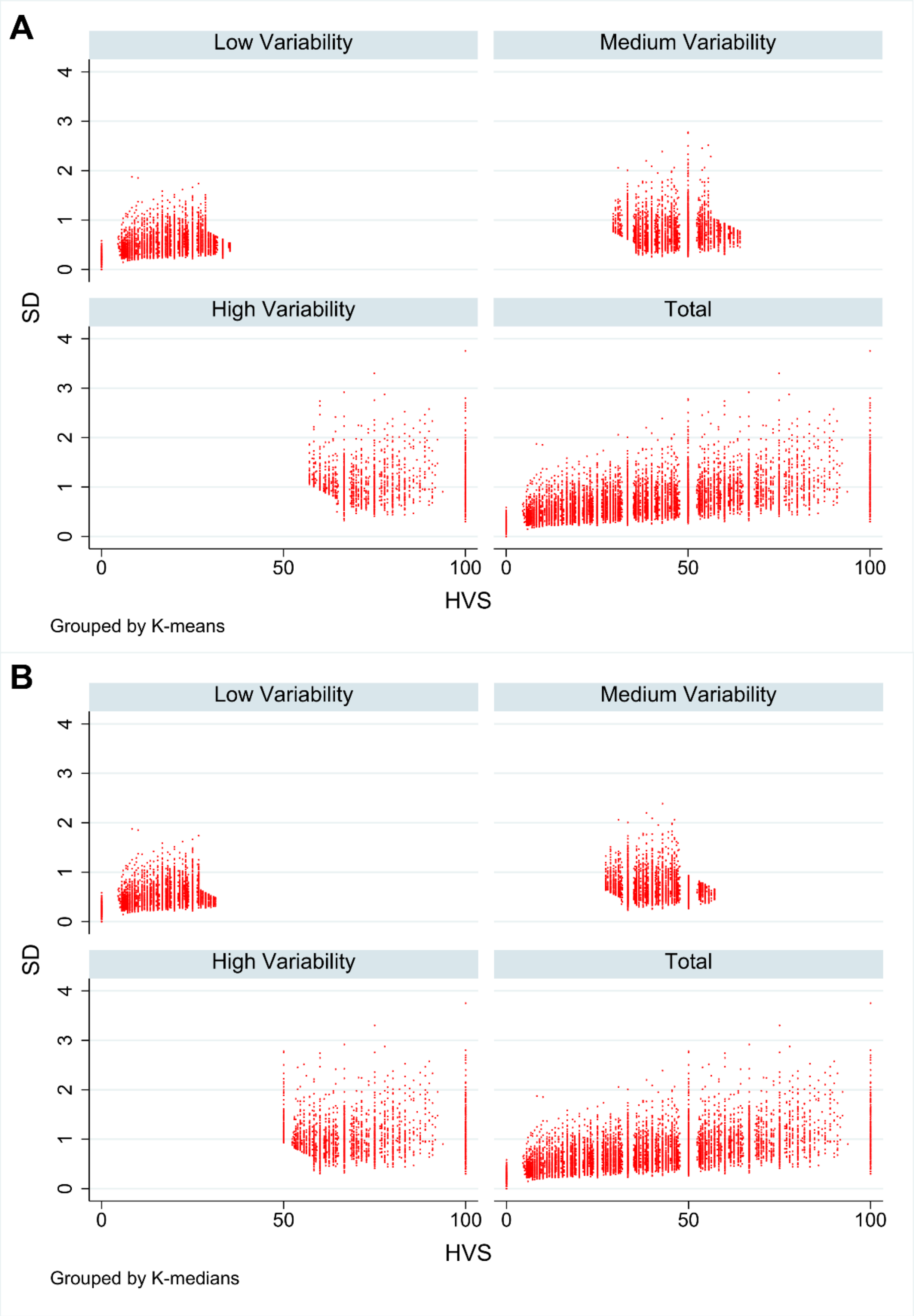

Figure S1. Clustering results for each algorithm.
A. Grouping by K-means. B. Grouping by K-medians.

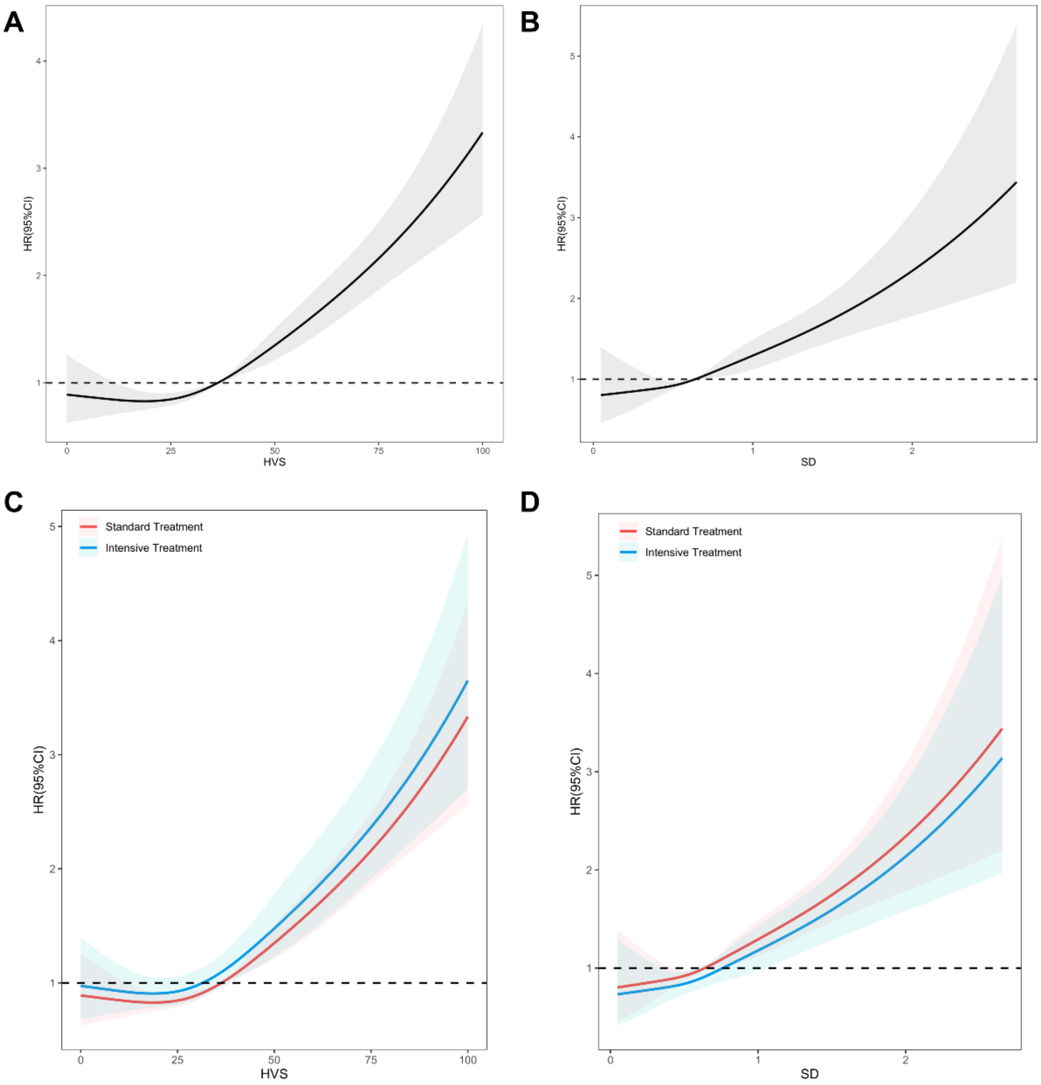

Figure S2. Relationship between HVD, SD, and primary outcome.
The solid line is the hazard ratio, the region is 95%CI.

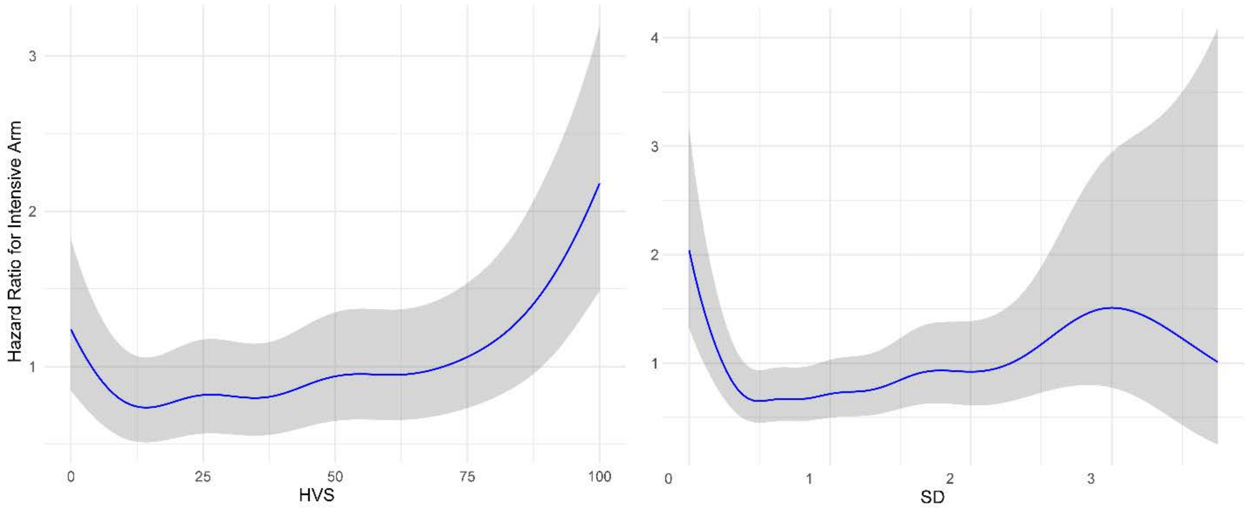

Figure S3. The effect of intensive *vs.* standard blood glucose management across the spectrum of HVS and SD for MACEs.

The solid blue lines represent the hazard ratio at each baseline HVS and SD interval. The grey range represent the upper and lower bounds of the 95% confidence interval.

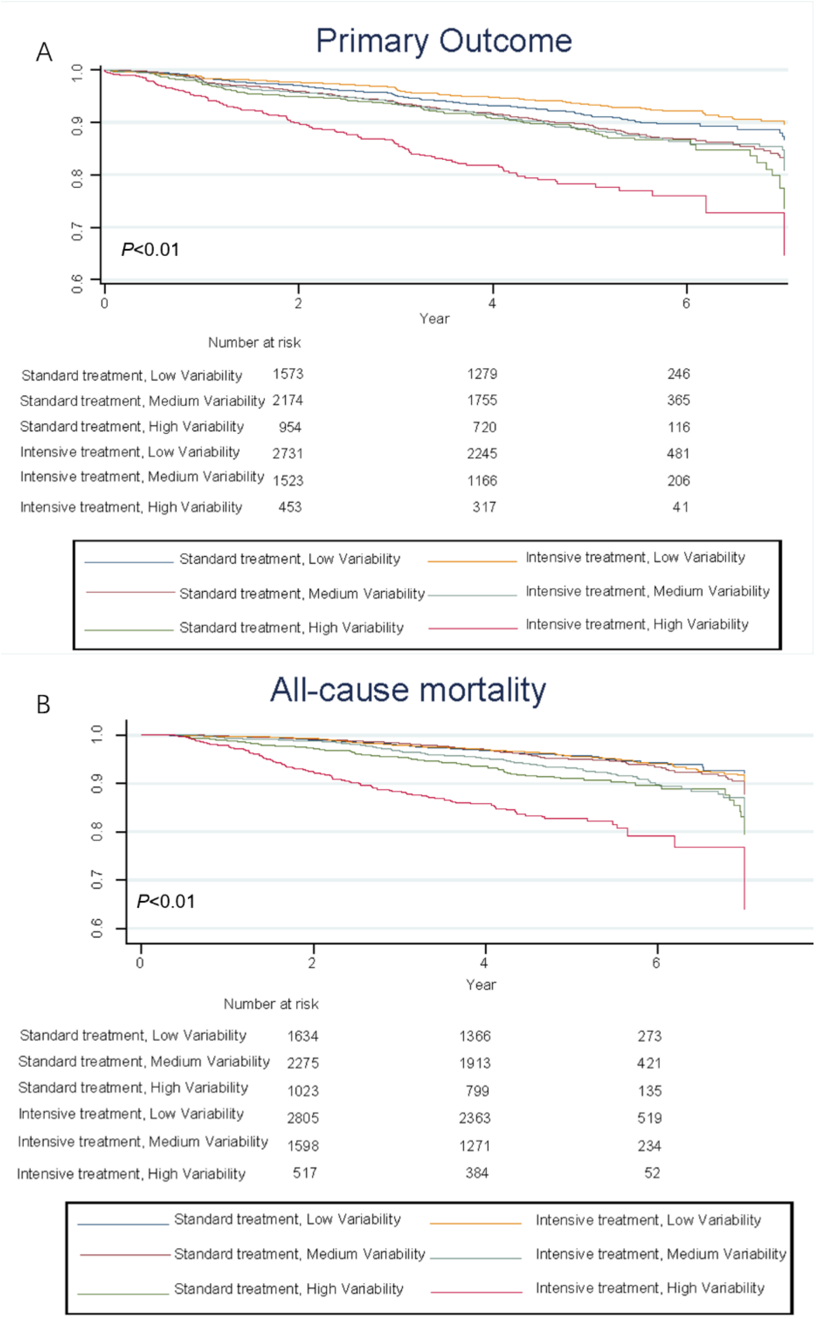

Figure S4. Kaplan-Meier survival curve for the primary outcome and all-cause mortality grouped by intensive treatment and HbA1c variability levels.

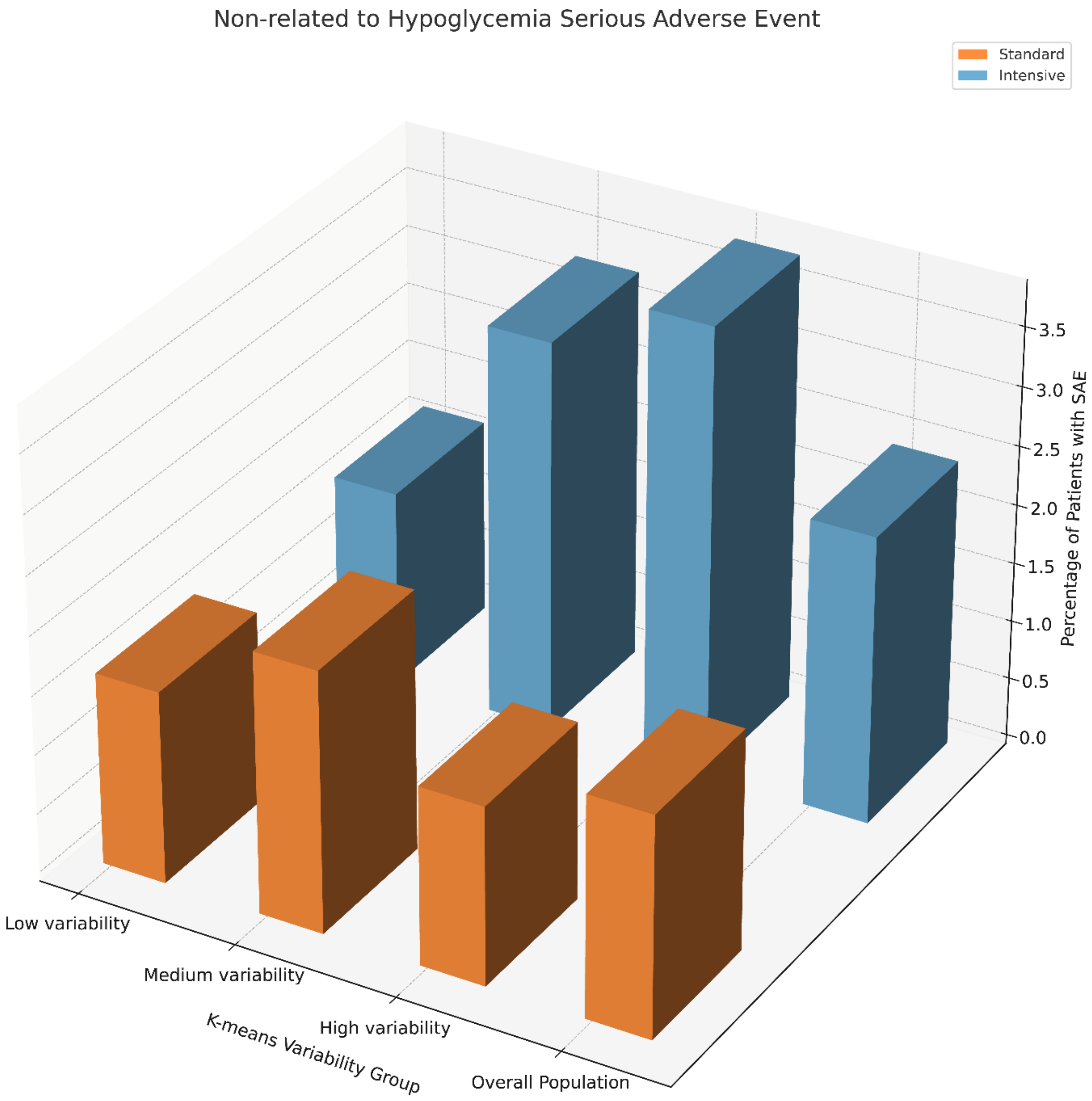

K-means Cluster	Low Variability	Medium Variability	High Variality	Overall population	*P* Value*
Standard arm	27 (1.62%)	51 (2.21%)	16 (1.51%)	94 (1.87%)	0.25
Intensive arm	47 (1.66%)	54 (3.31%)	22 (3.81%)	123 (2.44%)	<0.01
P Value#	0.46	0.02	<0.01	0.02	0.02$

Figure S5. The non-related to hypoglycemia serious adverse event in participants randomized to the intensive compared with the standard blood glucose treatment group, stratified by HbA1c variability levels.

* Difference among variability group,

Standard arm *vs.* Intensive arm,

P value was calculated by Fisher's exact z-test.

$ *P* for interaction trend test.

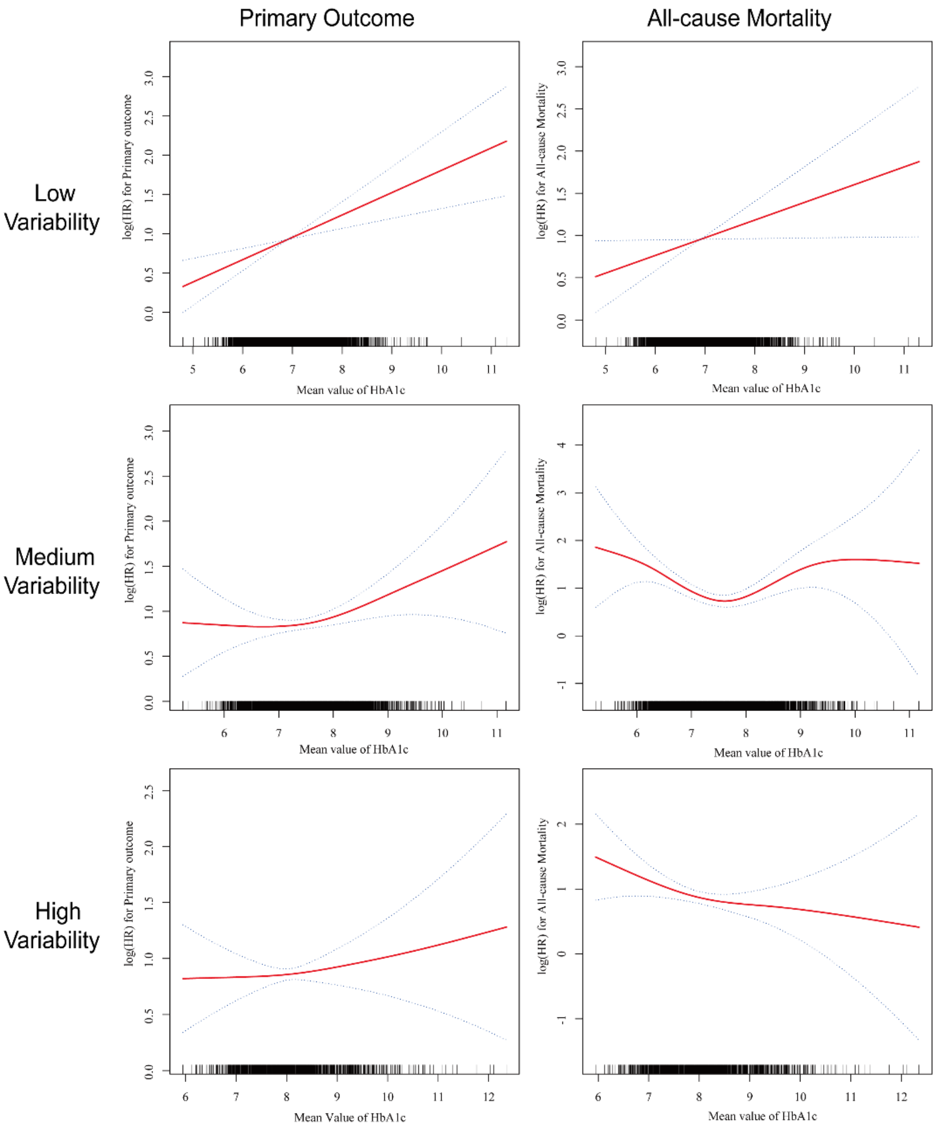

Figure S6. Association between the mean HbA1c value from all visits and the risk of primary outcome and all-cause mortality stratified by HbA1c variability.
The solid red line represents the estimated hazard ratio, while the dotted blue line indicates the 95% confidence interval.
